# Nutraceuticals in the Management of Intestinal Inflammation—Current Advances and Future Challenges

**DOI:** 10.3390/nu17071171

**Published:** 2025-03-28

**Authors:** Charalampia Amerikanou, Anastasia Z. Kalea, Efstathia Papada

**Affiliations:** 1 Department of Nutrition and Dietetics, School of Health Science and Education, Harokopio University, 17671 Athens, Greece; camer@hua.gr; 2 Division of Medicine, University College London, London WC1E 6JF, UK; a.kalea@ucl.ac.uk

Gut immunity begins developing in the early years in humans and is crucial in the maintenance of gut health in adult life. Gut dysbiosis is a hallmark of various gastrointestinal diseases, such as inflammatory bowel disease (IBD) and other chronic disorders, which pose significant health challenges as they can significantly impact the quality of life of millions of patients worldwide [1]. Conventional treatments often involve corticosteroids and immunosuppressants, which can be effective, but they are frequently associated with adverse side effects, particularly after long-term use, and may not provide long-term relief.

In recent years, nutraceuticals have emerged as a promising complementary approach in managing intestinal inflammation. Nutraceuticals include a wide range of foods or food components, such as isolated nutrients, herbal products, dietary supplements, novel foods, and processed food ingredients that provide medical or health benefits. They offer several biological properties, such as antioxidant, antihypertensive, immunomodulatory, antiproliferative, antimicrobial, and anti-inflammatory activities, while their natural origin and multifaceted health benefits make them attractive candidates for managing chronic inflammatory conditions. However, the mechanisms by which nutraceuticals exert their anti-inflammatory effects are complex and often not fully elucidated. Polyphenols, for example, exert their effects by scavenging free radicals and downregulating inflammatory pathways while strengthening gut barrier function. Omega-3 fatty acids are known to inhibit the production of pro-inflammatory cytokines and eicosanoids, which play a key role in the inflammatory cascade while actively converting to specialised pro-resolving mediators of the inflammatory response and inhibiting the activation of nuclear factor-kappa B (NF-κB). Probiotics can alleviate inflammation via the enhancement of intestinal barrier function and the modulation of the immune response while also producing natural antimicrobial peptides that inhibit pathogen growth [2].

The intricate relationship between nutraceuticals and intestinal health has attracted significant attention in recent years, particularly in the context of nutraceuticals with potential therapeutic benefits. This Special Issue brings together novel research studies that explore various nutraceuticals and their effects on intestinal health and inflammation, offering insights into potential treatments for gastrointestinal disorders and other related conditions.

Gut immunity development begins in the early years, and the role of gut microbiome in intestinal health is pivotal from infancy. Dietary supplements, prebiotics, and probiotics may exert important immunoregulatory effects on the gut immune system. Immunoglobulin A (IgA) is one of the most important types of antibodies in early life that protects mucosal integrity and plays a key role in the development of the intestinal mucosal immune defence system. For their article in this Special Issue, Ding and colleagues [3] investigated the potential of different strains of *Bifidobacterium longum*, a species with established immunoregulatory activities, to regulate IgA levels in newborn mice. Pathogen-free BALB/c mice were gavaged with either normal saline or different *Bifidobacterium longum* strains, and then, at 3 weeks old, IgA and secretory IgA (sIgA) levels were measured in the colon. A 16S rRNA gene-sequencing analysis was also performed in faecal samples. Five strains were found to increase IgA or sIgA levels, and, interestingly as regards the gene expression of IgA-related genes, several upregulations were observed in the colon and the ileum. Some strains induced a significant increase in the Chao 1 and Shannon Index only in male mice and also lowered the relative abundance of *Escherichia coli*. These results suggest that specific probiotics may offer immunoregulatory effects, not only contributing to the upregulation of IgA and related genes but also affecting the abundance of other bacteria.

Moving from infancy to adulthood and older age, the composition of the gut microbiota changes significantly. Gut dysbiosis may contribute to low-grade chronic inflammation, oxidative stress, and the production of various toxic metabolites, as well as decreased gut barrier function and reduced immunity, leading to an increased risk of chronic diseases [4]. Liu and colleagues [5] investigated the effects of selenium- and/or zinc-enriched eggs (SZEs) in ameliorating oxidative stress injury, alleviating cognitive impairment, and maintaining intestinal gut microbiota balance using a D-gal-induced ageing mice model. Interestingly, SZE restored learning and memory abilities while increasing antioxidant levels and decreasing inflammation. Additionally, SZE maintained the gut microbiota balance and increased the abundance of *Firmicutes* and *Blautia*. *Blautia*, as a probiotic, correlated negatively with pro-inflammatory factors and positively with antioxidant levels. These results suggest that nutraceuticals may also play a role in preventing age-related decline, potentially mediated by the gut microbiota and effects on the intestinal barrier.

As well as their role in maintaining gut immunity and a healthy intestinal barrier, nutraceuticals may also contribute to the management of chronic gastrointestinal diseases such as inflammatory bowel disease. Olate-Briones and colleagues [6] investigated the anti-inflammatory effects of Yerba Mate (YM) (Ilex paraguariensis), which is a natural herbal supplement, by using the dextran sodium sulphate (DSS)-induced acute colitis model in mice. They evaluated the effect of YM on colitis symptoms and macrophage polarisation and showed a reduction in colitis symptoms and improvement in animal survival. More specifically, YM promoted an immunosuppressive environment via the increased infiltration of anti-inflammatory M2 macrophage in the colon of mice treated with YM and the promotion of M2 macrophage differentiation in vivo. YM also induced favourable effects on the gut microbiota, explaining in part the beneficial properties of this medicinal plant since controlling dysbiosis and stimulating the presence of favourable bacteria could improve colitis symptoms.

Another study using an animal model of DSS colitis investigated the effect of lactate, a major component of fermented foods (e.g., pickled Chinese cabbage and yoghurt) also produced by lactic acid bacteria in the gut, on intestinal barrier function [7]. Lactate plays a key role in modulating inflammation via G-protein-coupled receptor 81 (GPR81); thus, the authors aimed to investigate the potential role of GPR81 in the progression of colitis and to assess the impact of lactate/GPR81 signalling on intestinal epithelial barrier function. The results demonstrated a suppressive effect of lactate on tumour necrosis factor- alpha (TNF-α)-induced matrix metalloproteinase-9 (MMP-9) expression and the prevention of the disruption of tight-junction proteins by inhibiting NF-κB activation through GPR81 in vitro. Additionally, dietary lactate preserved intestinal epithelial barrier function against DSS-induced damage in a GPR81-dependent manner in vivo, highlighting the significant involvement of the lactate/GPR81 signalling pathway in maintaining intestinal epithelial barrier function.

Another gastrointestinal disorder where diet has been proven to play an essential role is irritable bowel syndrome (IBS). When first-line dietary measures are not effective, the low-fermentable oligosaccharide, -disaccharide, -monosaccharide, and -polyol (low-FODMAP) diet is recommended in diarrhoea-predominant irritable bowel syndrome (IBS-D). Chojnacki and colleagues [8] examined whether the combination of a low-FODMAP diet with a reduction in tryptophane intake may have a positive impact in IBS-D patients. The supplementation of tryptophan as a nutraceutical is associated with improved mood [9], since it is a precursor to serotonin. However, interestingly, this study followed a reducing approach in tryptophan intake, as, apart from its effects on mood, serotonin as a neurotransmitter strongly stimulates motility and intestinal secretion, and its excess exacerbates chronic diarrhoea and abdominal pain [10]. In the study by Chojnacki and colleagues [8], 80 patients with IBS-D were randomised into two groups and followed either the low-FODMAP diet or a combination of this diet with limited tryptophan intake for 2 months. Significant reductions in both physical and mental symptoms were observed after both interventions, but the group with limited tryptophan intake experienced greater improvements. Although the levels of tryptophan did not differ after either of the interventions, its urinary metabolites 5-hydroxyindoleacetic acid, kynurenic acid, and quinolinic acid were reduced only in the limited-tryptophan-intake group. These results support the hypothesis that, although tryptophan as a nutraceutical can positively affect mental health and mood, reducing its content in a low-FODMAP diet may enhance the diet’s effectiveness, which may be associated with the observed alterations to the kynurenine pathway and its possible implication in the serotonin pathway.

The potential of nutraceuticals in managing intestinal inflammation is vast, but several challenges remain. The lack of standardisation and regulation in the nutraceutical industry can result in variability in product quality and potency. Hence, more research is needed to determine the optimal dosages and long-term safety characteristics of these compounds. Future large-scale clinical trials should focus on validating the efficacy of these compounds and exploring their interactions with conventional treatments. Advances in personalised nutrition could also pave the way for tailored nutraceutical interventions based on individual genetic and microbiome profiles.

In conclusion, nutraceuticals represent a promising approach to the maintenance of gut immunity and the management of intestinal inflammation. Their natural origin and beneficial biological properties make them attractive candidates for use in an adjunctive therapy in gastrointestinal and other chronic diseases. As the research in this field continues to progress, nutraceuticals may become key components of holistic approaches to gastrointestinal health, providing patients with safer and effective options for managing chronic inflammatory conditions.
